# From Personal Trouble to Public Issue: Early-Onset Colorectal Cancer Among Young Latino Men

**DOI:** 10.7759/cureus.112022

**Published:** 2026-07-03

**Authors:** Hansel A Aguilar Avila, Jaime U Aguilar, Carmen V Martinez Espino

**Affiliations:** 1 Sociology and Anthropology, George Mason University, Fairfax, USA; 2 Independent Patient Advocate, Washington Metropolitan Area, USA; 3 Independent Community Organizer, Northern California, USA

**Keywords:** early-onset colorectal cancer, health inequities, latino health, sociological imagination, structural determinants of health

## Abstract

C. Wright Mills' concept of the sociological imagination connects personal troubles to public issues. This editorial, co-authored by a sociologist, a Latino cancer survivor, and a community organizing director, applies that framework to early-onset colorectal cancer (EOCRC) among young Latino men, where molecular epidemiology and structural inequities converge. Recent genomic research identifies colibactin mutational signatures disproportionately present in EOCRC tumors, suggesting early-life microbial exposures may shape later carcinogenesis. Yet biological evidence alone cannot explain why young Latino men bear heavier diagnostic delays, lower screening rates, and worse outcomes. Drawing on co-author Aguilar's diagnosis at age 38, co-author Martinez Espino's community organizing practice, and the published literature on Latino cancer disparities, this editorial argues that fully understanding EOCRC requires interdisciplinary integration of molecular and sociological evidence. Addressing these inequities demands culturally tailored prevention, expanded community health worker and patient navigation programs, and structural policy reform. Effectively integrating epidemiology with health equity and community-based knowledge offers a path to address EOCRC inequities more comprehensively than any single discipline alone. EOCRC among young Latino men is not only a biological phenomenon but a social epidemiologic issue demanding a collective response.

## Editorial

This editorial brings together three voices in scholarly collaboration: a sociologist (Aguilar Avila HA), a Latino man diagnosed with early-onset colorectal cancer at age 38 (Aguilar JU), and a health equity organizing director working with Latino families in the Bay Area (Martinez Espino CV). We write together because the topic demands it. Early-onset colorectal cancer (EOCRC) among young Latino men cannot be understood through any single disciplinary lens; it requires the integration of molecular epidemiology, lived experience, and community-based knowledge.

Mills argued that the sociological imagination connects personal troubles to public issues [1]. Epidemiology shares this orientation, linking individual outcomes to population determinants. A colorectal cancer diagnosis in a man in his thirties may appear isolated, yet rising EOCRC, particularly among Latino populations, reveals a public issue requiring structural analysis.

Colorectal cancer is now one of the leading causes of cancer death among adults younger than 50 [2], and emerging evidence suggests that Latino individuals may be disproportionately affected by young-onset rectal cancer [3]. Recent genomic analyses identify colibactin, a genotoxic bacterial toxin produced by certain strains of Escherichia coli, leaving mutational footprints disproportionately present in EOCRC tumors [4,5]. These signatures appear over three times more frequently in patients under 40 than in patients over 70, and emerge early in tumor development, a pattern consistent with organoid experiments suggesting colibactin's mutagenic effect on colonic epithelium [4,5]. The implication is striking: childhood exposures may leave mutational scars that surface decades later as malignancy.

But molecular signatures cannot, alone, explain the disproportionate EOCRC burden among young Latino men. Early-life microbiome disruption and dietary exposures shape risk [6,7], and these exposures are not randomly distributed. They cluster within populations shaped by migration histories, economic precarity, occupational hazards, and constrained access to preventive care. To understand EOCRC fully, the molecular evidence must be interpreted through the structural conditions that determine who is exposed, who is diagnosed early, and who survives. 

Latino populations in the United States are heterogeneous, encompassing diverse national origins, immigration histories, generational statuses, and regional contexts. These differences shape exposure patterns, healthcare access, and screening experiences in ways that defy single-narrative framings. While this editorial focuses on patterned disparities common across Latino men, future research must examine variation within this population to design more responsive interventions.

Biography as evidence: a co-author's diagnostic journey

Co-author Aguilar was diagnosed at age 38 with Stage 3A colorectal cancer. His diagnostic experience is contributed here as authorial testimony, not as a case report, and illustrates how individual journeys reflect patterned inequities documented across the literature.

In his own words: "Navigating the healthcare system became a full-time job. I had to advocate for myself, push back on what I suspected was potential malpractice, and coordinate my care between multiple providers. It felt like I was the case manager for my own health, ensuring my primary care provider sent information to the gastroenterologist and surgeon, making sure the surgeon's office completed paperwork for my insurance and employer, and confirming the hospital had everything it needed to schedule my surgery."

His symptoms began with abdominal discomfort that initially seemed unremarkable. An urgent care visit produced a referral to the emergency room but no diagnostic workup. Only when his primary care physician ordered a stool test and a CT scan did the diagnostic pathway open, eventually leading to a colonoscopy and the cancer diagnosis. The gastroenterologist initially resisted the colonoscopy request. As he recalls: "My hope was almost shattered when the GI doctor pushed back against my request for an endoscopy and colonoscopy. I was determined not to leave that office without scheduling the procedures."

The questions he asked himself during that period are not merely personal. They are diagnostic of the structural conditions documented in the literature: "Was I being dismissed because I was young? Was it because I am Latino? Was it because my BReast CAncer (BRCA) inherited gene was not considered to correlate with colorectal cancer?" These questions echo broader research demonstrating that Latino patients experience diagnostic delays due to insurance gaps, language barriers, and cultural stigma [8]. Hispanic populations in the United States remain among the least insured; in 2014, 26.5% of Hispanics under age 65 lacked insurance compared to 10.4% of non-Hispanics [9]. Masculinity norms further shape health-seeking behavior, with qualitative studies showing that fear, embarrassment, and low perceived susceptibility discourage Latino men from colorectal cancer screening [8].

His reflection on what should change reads as a call for structural reform: "A person's age, race, or medical history should never be a barrier to care; they should be indicators. We shouldn't have to wait until symptoms start to make an appearance; we should make it a standard practice to ask for a stool test, an endoscopy, a colonoscopy, or any other relevant exam." This is biography speaking the language of population health. It is also why his name appears on this editorial as a co-author rather than as a case under study.

From individual troubles to collective response: an organizer's perspective

If biography reflects history, then individual stories of diagnostic delay reflect collective failures of healthcare structure. Co-author Martinez Espino works as a health equity organizing director with Latino families in the Bay Area, where the gap between healthcare need and healthcare access is daily and visible. Her contribution to this editorial reflects practice-based knowledge developed through years of community organizing on cancer access, screening, and survivorship.

Recent federal policies have further restricted access to healthcare for marginalized communities, compounding long-standing inequities in coverage and care. As Martinez Espino observes in her organizing work, community-based organizations translate individual suffering into collective response. Survivorship alone is insufficient when the conditions producing late diagnosis remain unchanged. Community organizing and participatory approaches have been shown to strengthen power among marginalized populations, leading to measurable policy outcomes and advances in health equity [10-12].

The Latino Health Assessment in Santa Clara County, California, documents that Latino communities face systematic barriers to accessing care, including language and communication challenges, insurance confusion and bureaucracy, delays in care, deep distrust in quality of care, widespread misinformation and stigma around illness, cost concerns, and delayed screenings [13]. These are not individual challenges. They are systemic conditions, public issues in Mills's sense.

One community member quoted in the Santa Clara assessment captured the cost of these failures directly: "I had cancer last year, and I did not qualify for many aids. And those that I did qualify for were not in Spanish. They translated for me, and it was horrible. A translator is not a way to receive or communicate expressions. They told me I was in Stage 4 when in reality, it was Stage 2" [13]. A translation error of two cancer stages is not an interpretive nuance. It is a structural failure with potentially fatal consequences.

In Martinez Espino's organizing practice, the Promotoras Model engages trained community members with firsthand experience to deliver education in culturally and linguistically appropriate ways. This approach empowers community members, often patients themselves, to dispel myths and misinformation, build trust, and foster open dialogue that supports informed health decisions and improved outcomes. Evidence supports community health worker (CHW) and patient navigation programs as effective interventions to increase colorectal cancer screening in Latino communities [14-16]. Mailed fecal immunochemical testing combined with navigation has been shown to increase screening participation and reduce disparities [17]. Yet current guidelines begin average-risk screening at age 45 [18], leaving younger symptomatic men, including patients like co-author Aguilar, vulnerable to precisely the diagnostic delays documented in both individual experience and the broader literature.

Integrating biology, biography, and structural context

Colibactin signatures highlight how early-life exposures shape cancer risk, but these exposures are inseparable from migration, economic precarity, and environmental injustice. At an ecosystem level, Latino populations are disproportionately exposed to environmental hazards including air pollutants [19], food deserts, and occupational risks that operate not as discrete factors but as interconnected conditions of daily life. These exposures interact with biological vulnerabilities to reinforce inequities. The molecular and the structural are not competing explanations for EOCRC; they are interdependent dimensions of the same phenomenon.

Interdisciplinary integration demands that epidemiology recognize sociological determinants as co-equal with molecular findings. Recent sociological frameworks, including White's relational approach to wellbeing [20], reinforce this orientation by situating individual health within networks of relationships, structures, and conditions rather than isolated biological factors. Without this, research risks reinforcing biological determinism while overlooking the systemic inequities that determine which biological vulnerabilities translate into late diagnoses and worse outcomes.

Toward equity-driven prevention and policy

Three priorities emerge from this analysis. First, interdisciplinary approaches must integrate molecular epidemiology with structural determinants of health to understand EOCRC risk among Latino populations comprehensively. Second, culturally tailored screening strategies, including investment in community health worker and patient navigation programs, must expand to address the diagnostic delays documented in both individual experience and the broader literature. Third, policy-level interventions must mitigate the environmental and occupational exposures that disproportionately affect Latino communities and contribute to cumulative cancer risk.

The rise of EOCRC among young Latino men exemplifies Mills's call to connect personal troubles to public issues. A survivor's contribution to scholarship is not anecdote but evidence of patterned disparities. Colibactin research uncovers biological risks, but inequitable structures explain why some populations bear heavier burdens. The collaboration represented in this editorial enacts the kind of interdisciplinary integration the topic demands. A sociologist alone could not have written it. A survivor alone could not have written it. An organizer alone could not have written it. Together, drawing on disciplinary training, lived experience, and community practice, we have written one editorial that argues for what EOCRC research, clinical care, and public health policy must become. From molecular footprints to social footprints, the path forward requires that we treat EOCRC not only as a biological phenomenon but as a social epidemiologic issue demanding collective response.
